# ﻿The Aranzadi bird Ringing Scheme data bank

**DOI:** 10.3897/zookeys.1238.136941

**Published:** 2025-05-09

**Authors:** Juan Arizaga, Agurtzane Iraeta, Ariñe Crespo, Francisco Pando

**Affiliations:** 1 Aranzadi Ringing Scheme, Aranzadi Sciences Society, Zorroagagaina 11, 20014 Donostia, Spain Aranzadi Sciences Society Donostia Spain; 2 Real Jardín Botánico-CSIC, Madrid, Spain Real Jardín Botánico-CSIC Madrid Spain

**Keywords:** Avian research, banding, biological data management, citizen science, EURING, ring-recovery data, Spain

## Abstract

The Aranzadi Ringing Scheme (ARS), operated by the Aranzadi Sciences Society, is an official bird-ringing program in Spain. Established in 1949, the data bank of the ARS is published, with the data aggregated to some extent, in the Global Biodiversity Information Facility (GBIF). It is a dataset covering the period from 1950 to nowadays, although ringings—but not recoveries—up to the 1970s remain, in part, to be digitalized. Ringings are carried out in Spain, and only exceptionally in third countries where there is not an official, operative ringing scheme. Recoveries of birds with Aranzadi rings can be potentially collected elsewhere; currently, recoveries of birds have been on all the continents—but not in Oceania and on Antarctica—with in the bounding coordinates of 59.0°N to 33.8°S and 62.8°E to 33.8°W, but > 90% of the records are within Europe. Up to 31 December 2024, the dataset includes 1.8 million records of either ringings or recoveries, all of which are georeferenced. In total 479 taxa are included, of which 430 are species. The rest are subspecies, hybrids, or birds identified only to genus. Twenty-four orders are represented by the data.

## ﻿Introduction

Bird-ringing datasets constitute a important source of temporal and spatial information on vertebrate taxa available worldwide (Harnos et al. 2015). Ringing schemes have the responsibility to (1) provide officially recognised metal rings to ringers in order to individually mark wild birds under scientific contexts, (2) keep stock of these rings, and (3) maintain the data from those rings in the long-term. This management also includes the exchange of ring-recovery data between schemes (Baillie 2001).

The Aranzadi Ringing Scheme (ARS), operated by the Aranzadi Sciences Society, is an official bird-ringing program in Spain. It is also a member scheme of EURING (European Union for Bird Ringing; https://www.euring.org). The EURING code assigned to the ARS is ESA. The ARS has been active since its establishment in 1949.

### ﻿General description

The ARS Databank gathers data pertaining the “Aranzadi” rings (i.e. ESA rings), which includes both the ringings as well as the recoveries and recaptures. Additionally, data of recoveries which, belonging to birds ringed by other schemes, were recovered by ringers of Aranzadi or notified by third parties to the ARS.

Ringers are supported by Aranzadi through the provision of free rings, which are paid for, in part, with public funding. The mission of the ARS is to safeguard the dataset generated through this ringing activity. The ultimate owners of the data are the ringers, and therefore the type and amount of the data sent to and published in third-party databases are, to a reasonable extent, aggregated (for details see Methods) to avoid any possible conflict of interest with the aims of active projects. That means that the almost 1.8 million records included in the ARS Databank (as of 31 December 2023) have been condensed into approximately 476,000 records published in the Global Biodiversity Information Facility (GBIF).

The summarized (aggregated) databank published in GBIF is also sent to the Ministry of Environment of Spain and copied to the administrations of those territories where there are ringings and/or recoveries, as well as to EURING. The aim of the present article is to describe the Summarized Bird Ring-recovery Databank of the Aranzadi Ringing Scheme (ARS) published in GBIF.

### ﻿Project details

The project personnel of the Aranzadi Ringing Scheme are J. Arizaga (Head, since 2007), A. Iraeta (Technician), and A. Crespo (Technician). The data gathered in the ARS databank are the result of the fieldwork by hundreds of ringers, most of whom are volunteers. Previous heads of the ARS were (chronologically): J. Elosegi, J.M. Faus, J. Riofrío, and I. Zuberogoitia.

ARS staff salaries and other expenses relating to the functioning of the scheme ARS, including rings and their sending to ringers, data management, administration, coordination, and dissemination are covered by Public Basque Administrations: Basque Government and the Regional Councils (Deputations) of Álava/Araba, Bizkaia and Gipuzkoa (agreements), and supplemented with core funds of the Aranzadi Sciences Society. Volunteers participating in fieldwork cover their own expenses. This last cost largely exceeds (by several orders of magnitude) the cost of the ordinary functioning of the ARS.

### ﻿Geographic coverage

**Figure 1.**
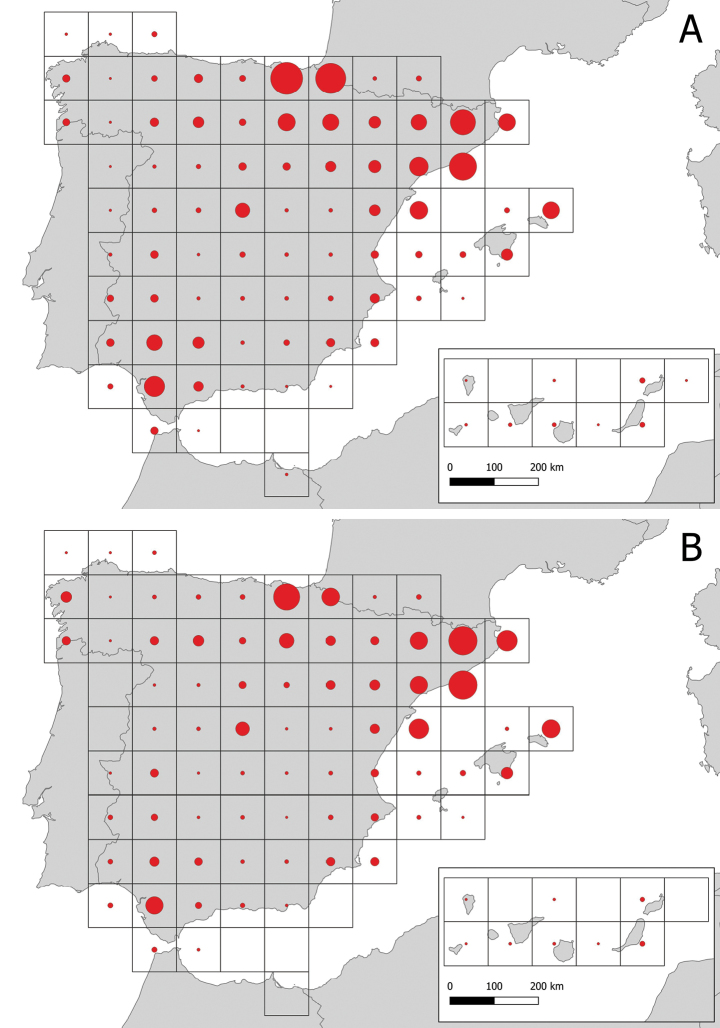
Ring-recovery data of Aranzadi (ESA) rings in Spain, lumped into UTM cells of 100 × 100 km **A** Ringings. Circle size refers to number of ringings (maximum size: 50,000 ringings) **B** Recoveries. Circle size refers to number of recoveries (maximum size: 15,000 recoveries).

**Figure 2.**
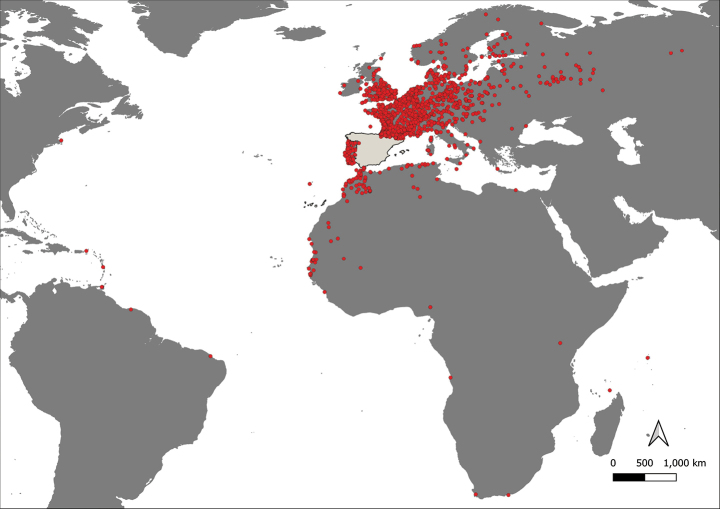
Geographical distribution of “Aranzadi” recoveries, outside Spain (shaded in pale grey).

Ringing is carried out in Spain, including the two archipelagos (Balearic Islands and the Canary Islands) and the two Spanish enclaves in North Africa (Ceuta, Melilla) (Fig. 1). Exceptionally, the Aranzadi rings are used in other countries where there is no official, operative ringing scheme. These rings, therefore, have been used in expeditions to Africa, in countries such as Morocco and Senegal (Arizaga et al. 2011; Arizaga et al. 2012). The ARS also provides service to Andorra. Recoveries of birds with Aranzadi rings can be potentially collected elsewhere. Currently, these recoveries cover Europe, Africa, Asia and America (Figs 1, 2).

**Bounding coordinates**: SW [33.8°S, 64.8°W], NE [69.0°N, 62.8°E].

### ﻿Taxonomic coverage

Birds. All kind of wild birds captured for ringing in the above mentioned study areas. 479 taxa (430 of them are species and the rest are mostly subspecies or, to a lesser extent, hybrids or birds with only known genus) belonging to 27 orders: Accipitriformes, Anseriformes, Apodiformes, Bucerotiformes, Caprimulgiformes, Charadriiformes, Ciconiiformes, Coliiformes, Columbiformes, Coraciiformes, Cuculiformes, Falconiformes, Galliformes, Gaviiformes, Gruiformes, Otidiformes, Passeriformes, Pelecaniformes, Phaethontiformes, Phoenicopteriformes, Piciformes, Podicipediformes, Procellariiformes, Psitaciformes, Pteroclidiformes, Strigiformes, Suliformes (Gill et al. 2025). This list of the species can be consulted at https://www.ring-eus/informes.

### ﻿Temporal coverage

**Period**: 1950-10-12 to 2024-04-08. Ringings (but not recoveries) up to the decade of 1970 remain in part to be digitalized. We estimate that these might involve at least 150,000 additional records.

## ﻿Methods

Data collection is carried out using the EURING code standards, i.e., controlled vocabularies as stated by EURING (Speek et al. 2001; Du Feu et al. 2020). There are more than 60 fields (for details see Du Feu et al. 2020) that, overall, fix the criteria to register data on the ringing scheme, ring code and the use of other marks, species, catching method, sex and age, date, time, geographical position (coordinates), several fields related with individual biological traits (morphology, moult, brood size, etc.), and the condition and circumstance of the encounter (e.g., ringed, shot, etc.).

Specific comments to be highlighted regarding methodological questions:

1. Species. Currently, both EURING and the ARS use the IOC (International Ornithological Congress) taxonomy.
2. For the specific case of the ARS databank, the data have been collected using different methods, depending on the nature and aims of each ringing project. Such methods include, among many other techniques (Bub et al. 1996), mist nets at constant effort sites working under standardized protocols (e.g., Arizaga et al. 2023), chicks captured by hand in their nests (e.g., in colonies of seabirds, raptors breeding either in cliffs or trees, etc.), spring traps baited with insects to capture some territorial small passerine birds, clap nets, etc. Overall, the data can belong to one of the following two main project type categories: (1) projects coordinated directly from the ARS, most of which are based on constant effort sites using standardized mist-netting (Ralph and Dunn 2004), or (2) specific projects, normally created and managed by individual ringers or groups of ringers, e.g. designed to monitor given species (e.g., long-term population dynamics survey protocols), to promote and enhance training capacities, to environmental education and formation, etc.
3. Currently, the coordinates are taken with great accuracy, but in the past the accuracy of a locality was normally of ± 5 km. Since birds are very mobile organisms, this low accuracy poses no major impact on the quality of the data bank, particularly for the analysis of long-distance movement patterns. However, it might have clear limitations for the analysis of ecological processes that operate at smaller spatial scales, including short-distance range dispersal or mark-recapture modelling.

Before inserting new data into the system, a series of filters are applied to guarantee a minimum quality of the data: (1) in all the fields having categorical nature (that is, values are chosen from a closed list of possible values), such as the species and catching method, the system automatically checks for the consistency of values; (2) the coordinates are examined to detect impossible values (e.g., offshore or at an impossible latitude or longitude); (3) in recoveries and recaptures, it is checked whether date was not before ringing date and whether the species identified when the bird was ringed was the same of that reported for the recovery.

**Figure 3.**
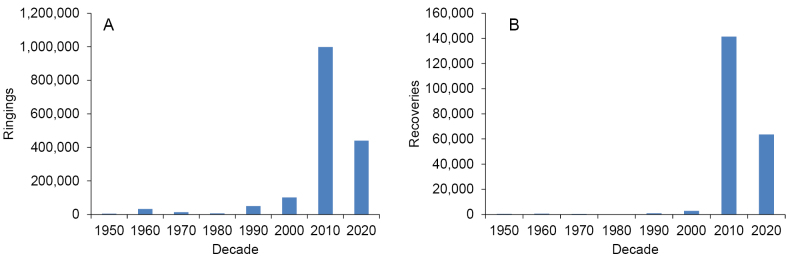
Cumulative number of ringings (**A**) or recoveries (**B**) existing within the ARS databank. Note that the series of the data ends, for analytical purposed, the 31/12/2024.

The ARS Databank contain records for ringing or recovery encounter, that is, each record corresponds to an individual bird. The ARS Databank contains 1,773,910 records (December 2023) and it is continually updated. Up to the decade of 1990, the mean number of ringings per decade was around 20,000 (but note that many ringings for the period 1950–1970 remain to be digitized; Fig. 3A). This pattern started to change in 2000, and definitively in 2010, with almost 1,000,000 ringings, due to the incorporation of many ringers that until then had been using the ‘Ministry of Environment’ rings from the Government of Spain. A similar pattern can be seen for recoveries (Fig. 3B). Up to 1990, the mean number of recoveries per decade was around 500. Note also that, in older times, ringers were obliged to register only long-distance (>10 km), long-temporal (>1 year) or foreign recoveries, so many within-site recoveries and recaptures were simply non-recorded within the ARS databank.

The summarised bird ring-recovery data set described here (i.e., the data set published in GBIF) derives from the ARS Databank through a data aggregation process. The ARS Databank records are combined in one record in the published dataset when they have the same values for the following parameters:

1. Date. Recorded under “day”, “month”, “year”, and “verbatimEventDate”
2. Species. Recorded using the Darwin Core terms for taxon.
3. Sampling protocol. Recorded under “samplingProtocol”, following the EURING code manual (Speek et al. 2001; Du Feu et al. 2020).
4. Whether the records correspond to a new ringing or to a recovery, provided under “dynamicProperties”
5. Condition (bird found alive, dead or sick or wounded), according to the EURING controlled vocabulary (Speek et al. 2001), provided under “dynamicProperties”
6. Circumstance of the encounter, according to the EURING controlled vocabulary (Speek et al. 2001), provided under “dynamicProperties”.

The number of records combined in each case is shown under the “dynamicProperties” field, as “Frequency”.

**Access link to GBIF**: https://www.gbif.org/es/dataset/52f2051b-c47e-403a-8e32-04b2f2273c20.

Partly, raw data are accessible for consultation and research under request, using an online form https://www.ring.eus. Requests are evaluated and decisions are taken by a committee representing the ringers. This committee is elected by ringers democratically, and renewed every two years.

**Object name**: Summarized Bird Ring-recovery Databank of the Aranzadi Ringing Scheme (ARS).

**Character encoding**: Darwin Core Archive UTF-8 (Wieczorek et al. 2012).

Publication date of data: updated annually. Data available since 1950.

**Language**: Spanish and English.

**Records**: up to 2024-04-08, almost 1,860,000 records, considering both the ringings and recoveries (for details see Table 1), all of which are georeferenced. Note, however, that most ringings before 1970 remain to be digitalized.

**Table 1.** Global stats of the ARS data bank, relative to ringings and recoveries of birds with “Aranzadi” rings.

Type of data	No. rings	No. species
Ringings	1,571,919	429
Recoveries	201,991	298

**Licences of use**: CC BY 4.0.

## ﻿Additional information

### Author ORCIDs

Juan Arizaga https://orcid.org/0000-0003-1911-4078

Francisco Pando https://orcid.org/0000-0002-6321-9398

### Data availability

All of the data that support the findings of this study are available in the main text.
